# Efficacy of amodiaquine in the treatment of uncomplicated falciparum malaria in young children of rural north-western Burkina Faso

**DOI:** 10.1186/1475-2875-7-58

**Published:** 2008-04-17

**Authors:** Germain Mandi, Frank P Mockenhaupt, Boubacar Coulibaly, Peter Meissner, Olaf Müller

**Affiliations:** 1 Centre de Recherche en Santé de Nouna, Nouna, Burkina Faso; 2 Institute of Tropical Medicine and International Health, Charité – University Medicine Berlin, Berlin, Germany; 3 Department of Tropical Hygiene and Public Health, Ruprecht-Karls-University Heidelberg, INF 324, 69120 Heidelberg, Germany

## Abstract

**Background:**

Combination therapy has become a new paradigm in malaria treatment. Amodiaquine is a common partner drug in different malaria combination therapies used or investigated in sub-Saharan Africa, but data on its efficacy as a single drug are scarce.

**Methods:**

The objective of the study was to determine the efficacy of amodiaquine against falciparum malaria in neighbouring rural and urban areas of north-western Burkina Faso. The study was designed as an uncontrolled trial in children aged 6–59 months with uncomplicated falciparum malaria in the Nouna Health District.

**Results:**

During the rainy season 2005, 117 children were enrolled, 62 from the rural and 55 from the urban study area. The crude adequate clinical and parasitological response (ACPR) rate was 103/117 (88%) by day 14 but decreased to 28/117 (24%) by day 28. After PCR correction for reinfections, ACPR rates were 108/117 (92%) and 71/117 (61%) by day 14 and day 28, respectively. There were no significant differences in efficacy between urban and rural areas. The *Plasmodium falciparum crt *K76T mutation not predict AQ failure, but was selected in parasites re-appearing following treatment. No serious adverse events occurred and only 16 other adverse events were recorded.

**Conclusion:**

Compared to chloroquine, amodiaquine is more effective against uncomplicated falciparum malaria in Burkina Faso. However, a considerable degree of amodiaquine resistance already exists and it is currently unclear how this resistance will develop when amodiaquine in combination with other drugs is used on a large scale.

**Trial registration:**

Current Controlled Trials ISRCTN73824458.

## Background

In sub-Saharan African (SSA), the basis of malaria control remains early diagnosis and prompt treatment with effective drugs [1]. This strategy is now complicated by increasing resistance of *Plasmodium falciparum *to existing and affordable first-line drugs such as chloroquine (CQ) or sulphadoxine/pyrimethamine (SP) in most SSA countries [1-3]. Combination treatment has become a new paradigm in malaria control, with the particular aim to delay and possibly reverse the development of drug resistance [4-9]. Artemisinin-based combination treatment (ACT) involving a variety of partner drugs has proved highly effective in a number of field trials [10-21]. However, the inexpensive combination of sulphadoxine/pyrimethamine *plus *amodiaquine (AQ) has recently been shown in a number of countries to be of comparable efficacy under the current epidemiological situation in SSA [10,19,22-28].

In Burkina Faso, *in vivo *CQ resistance was first reported in 1988 and clinical failure rates in children with uncomplicated malaria were around 5% in the early 1990s [29]. More recent data indicate that CQ in north-western Burkina Faso has gradually lost efficacy and now suffers treatment failure rates of partly more than 50% by day 14, with marked differences between rural and urban areas [30-32].

Amodiaquine has been reported to remain remarkably effective in many countries of SSA and in particular in West Africa despite emerging CQ resistance [11,27,33-36]. This has also been documented for north-western Burkina Faso [27,35,36]. It is thus considered as a useful partner drug in both, ACT and in non-ACT. In Burkina Faso, the first-line treatment against uncomplicated malaria has been changed in 2005 to ACT, including artesunate (AS)-AQ [37]. Moreover, the potential of AQ as a partner drug in methylene blue-based combination therapies is currently investigated in the Nouna Health District of Burkina Faso [31,38]. Against this background, the efficacy of amodiaquine monotherapy was tested in this study.

## Materials and methods

### Study area

The study was conducted from September to November 2005 in the research zone of the Centre de Recherche en Santé de Nouna (CRSN) in Nouna Health District, north-western Burkina Faso. The Nouna area is a dry orchard savannah, populated mainly by subsistence farmers of different ethnic groups. Malaria is holoendemic but highly seasonal with most cases occurring during or briefly after the rainy season which lasts from June until October [39]. Formal health services are limited to a few rural health centres and the district hospital in Nouna town (25,000 population). CQ as well as SP were shown to be still quite effective in the rural, but not in the urban CRSN study area [30,40]. Despite an official policy change from CQ to ACT as national malaria first-line therapy in early 2005, CQ has remained the main drug used for fever treatment in the area until today [37].

### Study design and objectives

The study was designed as an uncontrolled efficacy trial in two different areas (urban and rural) of Nouna Health District. The study was open label, with blinding only for the laboratory technicians being responsible for parasite determination in blood smears.

The primary objective of the study was to determine and compare the day 14 efficacy of AQ in the treatment of young children with uncomplicated falciparum malaria in the rural and urban CRSN study area. Secondary objectives were to determine and compare the day 28 efficacy of AQ in the rural and urban CRSN study area and to determine the safety of AQ in the treatment of young children with uncomplicated falciparum malaria in Burkina Faso.

### Patients and study procedures

After information of the study communities, mothers with febrile young children were invited for examination by the study team to specific fever measurement points (public buildings). Out of 420 children screened, a total of 117 children were enrolled, 62 from two purposely (good accessibility) chosen villages in 10 and 20 km distance from Nouna town respectively, and 55 from two randomly chosen city quarters of Nouna town. It was initially planned to include 120 children, 60 from the urban and 60 from the rural study area. This sample size would allow for a 20% loss to follow-up during the determination of an overall 40% failure rate (95% confidence level) at day 28 at a precision of 10%, as well as the detection of a major difference in AQ resistance between the rural and urban study areas.

Inclusion criteria were: age 6–59 months, uncomplicated falciparum malaria (axillary temperature ≥ 37.5°C and ≥ 2,000 *P. falciparum *asexual parasites per μl blood), and written informed consent given by the parents/caretakers. Exclusion criteria were complicated or severe malaria (repeated vomiting, seizures or other neurological impairment, haemoglobin < 7 g/dl or haematocrit < 21%), any apparent significant disease (e. g. pneumonia, meningitis, hepatitis, severe diarrhoea, measles, severe malnutrition), and malaria treatment with western drugs and/or antibiotics with antimalarial potency during last seven days except chloroquine.

Study children received a total dose of 25 mg/kg oral AQ divided over three days (first and second day: 10 mg/kg, third day: 5 mg/kg; AQ tablets or syrup, essential drug stock, Burkina Faso Ministry of Health). All patients were directly observed for at least 30 min following each treatment. If vomiting occurred once, treatment was repeated. In case of repeated vomiting, the participant was withdrawn from the study. Children having fever ≥ 38.5°C received a standard dose of paracetamol tablets (= acetaminophen; 10 mg/kg every six hours) until symptoms subsided. Follow-up of study children was for 28 days using a slightly simplified version of the latest WHO protocol on drug efficacy testing [41]: A finger-prick blood sample was taken on days 0, 2, 14 and 28. From this, malaria parasitaemia (thin and thick blood smears) as well as haematocrit values were determined using standard CRSN procedures [39]. All malaria slides were examined by two experienced laboratory technicians supervised by one of the investigators (BC). Asexual parasites were counted on thick blood films against 200 white blood cells (WBCs) and parasite density was calculated assuming an average WBC count of 10.000/μL. Slides were declared negative if no parasites were seen in 400 fields on the thick film. For quality control a 10% random sample of blood films is regularly cross checked at the laboratory of the Heidelberg School of Tropical Medicine [39]. Follow-up procedures were carried out through trained field workers who visited study children at follow-up days in their homes. Self-reported adverse events were registered through a standard questionnaire during follow-up visits.

In isolates obtained at recruitment and at treatment failure, the *P. falciparum crt *K76T mutation associated with resistance to CQ, and limitedly AQ [42-47], was determined at the Institute of Tropical Medicine and International Health in Berlin (Germany) by PCR-RFLP [42]. Isolates comprising both, mutant and wildtype alleles were considered mutant. Differentiation between recrudescence and re-infection took place through determination of *msp1 *and *msp2 *genotypes in paired filter paper blood samples from day 0 and day 14 as well as day 28 and from any other day after day 7 when a child had developed malaria according to standard methodology [48].

### Statistical analysis

Treatment outcome was categorized according to the latest WHO definitions for early treatment failure (ETF), late clinical failure (LCF), late parasitological failure (LPF) and adequate clinical and parasitological response (ACPR) [41]. Losses to follow-up and dropouts due to other reasons were considered as ETF or LCF depending on the time of dropout in an intention-to-treat manner.

The primary analysis parameter was the rate of clinical failures on day 14. Secondary parameters were the rate of clinical failures on day 28, the rate of early clinical failures, the rate of late parasitological failures (day 14 and day 28), and the rate of self-reported adverse events. Efficacy results were compared before and after PCR correction for reinfections. Data were analysed in the overall group of study children and for rural (n = 62) and urban (n = 55) study children separately.

The Chi square test (Chi) was used to compare rates, and exact binominal 95% confidence intervals (95%CIs) were calculated. The non parametric Wilcoxon-Mann-Whitney test (WMW) was applied to compare metric or ordinal data. The McNemar test was used to compare the proportions of *pfcrt *K76T at recruitment and at treatment failure. The statistical calculation was performed with SAS release 8.02 (SAS^® ^Institute Inc, Cary, NC, USA).

### Ethical aspects

The study protocol was approved by the local Ethics Committee in Burkina Faso.

## Results

### Study group characteristics

117 children with uncomplicated malaria were enrolled, 62 children from the rural and 55 from the urban study area, respectively (Table 1). The analysis is based on these 117 children. This is the *a priori *defined full analysis set (FAS) for the intention to treat analysis. There was no difference in the distribution of sex between the children from rural and urban area but children from the urban area were younger compared to children from the rural area (means, 26.4 months *vs*. 33.8 months, *P *< 0.001). Roughly one quarter of children had been treated for the current disease episode with CQ prior to inclusion, with no major differences between children of urban and rural residence. More children of the urban (63%) compared to the rural area (46%) carried parasites with the *pfcrt *K76T mutation (*P *= 0.06). There were six losses to follow-up, all but one were from the urban study area. The reason for the five urban cases was withdrawal by the parents who were dissatisfied with repeated blood taking. The reason for the rural case was a treated fever episode during follow-up without malaria parasitaemia.

**Table 1:** Baseline characteristics (D0)

Characteristic	Rural area (n = 62)	Urban area (n = 55)
Male	31 (50%)	25 (45%)
Age [months]		
- Mean	33.8	26.4
- Min, Max	6.0, 59.0	7.0, 48.0
Prior treatment of current disease episode with chloroquine	15 (24%)	17 (31%)
Haematocrit [%]		
- Mean	29.5	28.5
- Min, Max	22.0, 40.0	22.0, 36.0
*P. falciparum *trophozoites [/μl]		
- Median	16.000	18.000
- Min, Max	2.300, 175.000	2.000, 200.000
Fever (°C)		
- Mean	38.1	38.2
- Min, Max	37.5, 40.2	37.5, 39.5
*Pfcrt *codon 76 wildtype	33/61 (54%)	19/52 (37%)

### Safety of study drugs

There was no serious adverse event and no death over the 28 days follow-up period. Reported minor adverse events were 16 overall, eight in the rural and eight in the urban study children. Gastroenteritis was the main complaint in seven of the study children.

### Efficacy of study drugs

There was no ETF. The PCR-uncorrected rate of ACPR was 103/117 (88%) in the overall study group by day 14. This figure decreased to 28/117 (24%) by day 28. After PCR correction for re-infections, rates of ACPR were 108/117 (92%) and 71/117 (61%) by day 14 and day 28 respectively (Table 2 and Table 3). There were no significant differences in efficacy parameters between urban and rural study children.

**Table 2:** Treatment outcomes at D14

	Treatment outcome (No, %) [95 CI]		
	
	Rural area (n = 62)	Urban area (n = 55)	Total (n = 117)
ETF	0 (0%) [0–6%]	0 (0%) [0–6%]	0 (0%) [0–3%]
LCF			
- Recrudescence	0 (0%) [0–6%]	5 (9%) [3–20%]	5 (4%) [1–10%]
- New infection	0 (0%) [0–6%]	1 (2%) [0–10%]	1 (1%) [0–5%]
- No genotyping results	1 (2%) [0–9%]	0 (0%) [0–6%]	1 (1%) [0–5%]
LPF			
- Recrudescence	1 (2%) [0–9%]	1 (2%) [0–10%]	2 (2%) [0–6%]
- New infection	2 (3%) [0–11%]	2 (4%) [0–13%]	4 (3%) [1–9%]
- No genotyping results	1 (2%) [0–9%]	0 (0%) [0–6%]	1 (1%) [0–5%]
ACPR	57 (92%) [82–97%]	46 (84%) [71–92%]	103 (88%) [81–93%]
ACPR (pcr-corrected)	58 (94%) [84–98%]	50 (91%) [80–97%]	108 (92%) [86–96%]

**Table 3:** Treatment outcomes at D28

	Treatment outcome (No, %) [95 CI]		
	
	Rural area (n = 62)	Urban area (n = 55)	Total (n = 117)
ETF	0 (0%) [0–6%]	0 (0%) [0–6%]	0 (0%) [0–3%]
LCF			
- Recrudescence	9 (15%) [7–26%]	11 (20%) [10–33%]	20 (17%) [11–25%]
- New infection	5 (8%) [3–18%]	7 (13%) [5–24%]	12 (10%) [5–17%]
- No genotyping results	2 (3%) [0–11%]	2 (4%) [0–13%]	4 (3%) [1–9%]
LPF			
- Recrudescence	14 (23%) [13–35%]	5 (9%) [3–20%]	19 (16%) [10–24%]
- New infection	17 (27%) [17–40%]	14 (25%) [15–39%]	31 (26%) [19–35%]
- No genotyping results	2 (3%) [0–11%]	1 (2%) [0–10%]	3 (3%) [1–7%]
ACPR	13 (21%) [12–33%]	15 (27%) [16–41%]	28 (24%) [17–33%]
ACPR (PCR-corrected)	35 (56%) [43–69%]	36 (65%) [51–78%]	71 (61%) [51–70%]

### Association of treatment outcome with pfcrt K76T

*Pfcrt *K76T did not predict AQ treatment failure: children harbouring parasites with this mutation revealed a PCR-uncorrected (day 28) rate of ACPR of 26% (15/57) as compared to 25% (13/59) among children infected with wild-type parasites (*P *= 0.88; withdrawals excluded from analysis). Correction for re-infections did not change this finding (70%, 40/57 *vs*. 60% 31/52, *P *= 0.25). However, all (77/77) parasites isolated from children at AQ treatment failure exhibited the *pfcrt *K76T mutation as compared to 52% (40/77) at baseline (*P *< 0.0001). This was true for recrudescent infections (*n *= 33) and re-infections (*n *= 41; both, *P *= 0.0001).

## Discussion

The main finding from this study is that AQ is more effective than CQ in Burkina Faso but already affected by a considerable degree of drug resistance. CQ resistance has increased rapidly over the last couple of years in the Nouna Health District as in most of the country with more than 50 percent of all treatments in young children documented to have failed already by day 14 [31,37]. In comparison, AQ performs substantially better with a day 14 rate of APCR of 92 percent as observed in this study. This finding confirms the superiority of AQ in many SSA areas with intense CQ resistance [11,27,33,34,43]. However, AQ treatment was associated with a high rate of re-infections during follow-up, and by day 28, the PCR-corrected rate of APCR was reduced to 61 percent. This not only supports the importance of sufficiently long follow-up periods in antimalarial treatment trials [43] but also points to an established low-level AQ resistance in the area.

In urban western Burkina Faso, AQ achieves uncorrected and PCR-corrected cure rates of 82% [95%CI, 77–86%] and 92% [88–95%], respectively, during four weeks of follow-up [27], which clearly contrasts with the figures of 24% [17–33%] and 61% [51–70] observed in the current study. The present study had a limited sample size and, consequently, wide 95% confidence intervals, but this difference appears to not to be due to chance. However, at present, the reason for this discrepancy remains obscure. Aggravation of AQ resistance over time, population differences e.g. in access to (pre)-treatment, and varying transmission intensities might be involved. The vast majority of treatment failures in the present study occurred after day 14, and in roughly half of the failures, exclusively new parasite genotypes were detected indicating new infections. This proportion might even be higher, and consequently the actual cure rate better, considering a recent report illustrating that in highly endemic areas, a considerable number of new infections might be misclassified as recrudescences when applying conventional genotyping protocols [49]. In a previous trial in the study area, marked differences in the efficacy of CQ were observed between rural and urban settings with failure rates and the proportion of parasites exhibiting *pfcrt *K76T being higher in the latter [32]. This finding entailed the study design of the current trial. However, cure rates of AQ were observed to be even slightly higher in urban as compared to rural areas. Yet, this difference was marginal, the 95%CIs of the respective efficacy rates overlapped, and the present study had statistical power to detect only major differences. In the study area, the prevalence of *pfcrt *K76T is still relatively low, and similar to previous studies, *pfcrt *K76T did not predict AQ treatment failure but was selected among parasites re-appearing following treatment [44,45,47]. Recent evidence suggests particular *pfmdr1 *haplotypes of *P. falciparum *to be associated with AQ resistance [50]. In ongoing work, it is examined whether such could explain the observed unsatisfactory cure rate of AQ observed in the present study.

Efficacy and useful therapeutic life span of any antimalarial drug combination critically depend on the choice of the partner drugs and preexisting resistance patterns [12,51]. As AQ is still effective in most of SSA and as it is a drug considered sufficiently safe for malaria first-line therapy, it will be used both in ACT as well as in alternative malaria combination therapies. AS-AQ is now available as a fixed dose combination and has been chosen as one of two first-line malaria drugs for Burkina Faso [37]. Moreover, AQ was shown repeatedly in Burkina Faso as well as in other SSA countries to be very effective in combination with SP [10,19,22-28]. However, mainly for political reasons it was never employed in Burkina Faso despite such promising findings [37]. It is furthermore considered as a promising partner drug in combination with methylene blue [52]. However, AQ resistance exists in Burkina Faso and it is currently not clear how this resistance will develop when AQ in combination with other drugs is used on a large scale. Of particular concern, the combination of a high proportion of re-infections following AQ treatment and selection of a marker of drug resistance in treatment failures observed in this study reflects exposure of recrudescent and newly acquired parasites to low, subcurative AQ concentrations and might cause subsequent selection for resistance. This, in turn, would threaten the principle of mutual protection from resistance development in combination treatment.

## Conclusion

In this trial from north-western Burkina Faso, AQ treatment of children with uncomplicated malaria achieved an unsatisfactory cure rate of 61%. Impaired AQ efficacy, may shorten the useful life span of the first-line drug AQ-AS and of further AQ-based combination therapies. Continued monitoring of the efficacy of AQ is warranted.

## Competing interests

The authors declare that they have no competing interests.

## Authors' contributions

OM, GM and PM designed the study. GM, BC and FPM conducted the laboratory and clinical work. OM, GM and PM analysed the data. All authors contributed to the interpretation of the findings and to writing of the paper.
